# Importance of self-connections for brain connectivity and spectral connectomics

**DOI:** 10.1007/s00422-020-00847-5

**Published:** 2020-11-26

**Authors:** Xiao Gao, P. A. Robinson

**Affiliations:** 1 Department of Biomedical Engineering, University of Melbourne, Parkville, VIC 3052 Australia; 2 School of Physics, The University of Sydney, Sydney, NSW 2006 Australia; 3 Center of Excellence for Integrative Brain Function, The University of Sydney, Sydney, NSW 2006 Australia

**Keywords:** Brain connectivity, Eigenmodes analysis, FMRI, Neural field theory, Self-connections

## Abstract

Spectral analysis and neural field theory are used to investigate the role of local connections in brain connectivity matrices (CMs) that quantify connectivity between pairs of discretized brain regions. This work investigates how the common procedure of omitting such self-connections (i.e., the diagonal elements of CMs) in published studies of brain connectivity affects the properties of functional CMs (fCMs) and the mutually consistent effective CMs (eCMs) that correspond to them. It is shown that retention of self-connections in the fCM calculated from two-point activity covariances is essential for the fCM to be a true covariance matrix, to enable correct inference of the direct total eCMs from the fCM, and to ensure their compatibility with it; the deCM and teCM represent the strengths of direct connections and all connections between points, respectively. When self-connections are retained, inferred eCMs are found to have net inhibitory self-connections that represent the local inhibition needed to balance excitation via white matter fibers at longer ranges. This inference of spatially unresolved connectivity exemplifies the power of spectral connectivity methods, which also enable transformation of CMs to compact diagonal forms that allow accurate approximation of the fCM and total eCM in terms of just a few modes, rather than the full $N^2$ CM entries for connections between *N* brain regions. It is found that omission of fCM self-connections affects both local and long-range connections in eCMs, so they cannot be omitted even when studying the large-scale. Moreover, retention of local connections enables inference of subgrid short-range inhibitory connectivity. The results are verified and illustrated using the NKI-Rockland dataset from the University of Southern California Multimodal Connectivity Database. Deletion of self-connections is common in the field; this does not affect case-control studies but the present results imply that such fCMs must have self-connections restored before eCMs can be inferred from them.

## Introduction

A typical method to analyze brain connectivity is through connectivity matrices (CMs), which contain the strengths of connections between pairs of discretized regions of interest (RoIs), which are usually chosen to be functionally homogeneous and spatially contiguous; for example, RoIs can be chosen to be voxels in functional magnetic resonance imaging (fMRI), subdivisions of specific Brodman areas, or based on the subject’s own anatomy (Friston et al. 2006; Poldrack 2007). In CMs, the rows and columns in the matrices represent brain regions and entries represent the connections between brain regions (Bullmore and Sporns 2009; Sporns 2010; Friston 2011), so a CM is really a four-tensor that maps the 2D cortex to itself (Robinson 2019). The strengths of connection are defined in a variety of ways. Structural connectivity is often measured using anatomical CMs (aCMs), which summarize the density of axonal bundles between RoIs as measured by methods such as diffusion tensor imaging (DTI) and related diffusion-weighted magnetic resonance imaging (dwMRI) (Basser et al. 2000; Hofer and Frahm 2006). Functional connectivity is commonly measured via functional CMs (fCMs), which are commonly determined from the two-point covariance (equal-time correlations) of the activity in brain regions using fMRI, on the assumption that regions that are correlated are likely to be functionally related. Effective connectivity matrices (eCMs), sometimes termed gain matrices, quantify the actual effect of one brain region to another, including the strength of connections. The CMs that embody the strengths of direct connections between points in a given brain state are termed direct effective CMs (deCMs), whereas total effective CMs (teCMs) describe the total connectivity between points via both direct and indirect paths (Robinson 2012).

Analyses of CMs are highly topical. Previously, (Robinson 2012) showed how eCMs correspond to propagators, using neural field theory (NFT) (Galán 2008), and used the deCM to compute teCMs and fCMs. Later, the inverse problem of inferring the eCMs from fCMs was studied (Robinson et al. 2014), and eCMs were determined from the experimental fCMs of resting state brain activity using NFT and eigenfunction analysis. On the widely used assumption that the deCM is proportional to the aCM, the theory interrelated structural, functional, and effective CMs in terms of propagators (Robinson 2012; Robinson et al. 2014), which enabled the propagator theory to be applied to analysis of connectivity. The existence and strength of connections that were not detected using dwMRI were also inferred in this work, particularly interhemispheric links via the corpus callosum. Later, Robinson et al. (2016) applied NFT to predict and analyze the activity eigenmodes of the bihemispheric brain, focusing particularly on their spatial structure. The eigenmodes of a single brain hemisphere were found to be close analogs of spherical harmonics, which are the natural modes of a sphere. The results showed a close match to experimental brain connectivity data (Robinson et al. 2016).

Self-connections are commonly removed from experimental fCMs because they are thought to be trivial or because correlations are only computed between time series from different locations, i.e., the diagonal elements in the experimental fCMs are omitted or set to zero, as in many central papers in the field, such as Hagmann et al. (2008); Brown et al. (2012), and in the key examples cited from the literature in foundational texts, such as those by Sporns (2010) and Fornito et al. (2016). This does not affect case-control comparisons where the difference between CMs is examined directly and terms involving the self-connections cancel out. However, it is known that the diagonal entries in any normalized covariance matrix are all 1, by definition. In this paper, NFT is used to investigate how the deleted self-connections impact the properties and structure of CMs. In particular, by interrelating the fCM, teCM, and deCM using spectral analysis and NFT (Robinson 2012; Robinson et al. 2014), we investigate to what extent the missing self-connections in the fCM change the mutually consistent inferred eCM entries. First, an investigation of the importance of the self-connections in CMs is made. The role of the diagonal elements in the experimental fCMs is then clarified, the eCMs are calculated using NFT, and the effects of removing fCM self-connections on the inferred eCMs are demonstrated, showing that serious errors flow from failing to preserve the positive definiteness of the fCM, even though the proportion of connections omitted is tiny. The connectivity is also decomposed using eigenmode analysis, which enables representation of this modal brain connectivity in a compact diagonal matrix form and the rapid convergence is demonstrated (Robinson 2019).

It is worth stressing that the central aim of the present paper is *not* simply to prove that omission of diagonal entries changes the properties of the fCM in some way—that is entirely obvious (if you change any matrix you will change its properties). Rather, it is concerned with *how* the properties of the functional CM are changed and how these changes affect effective CMs inferred from the fCM via NFT. Second, it focuses on new results that can be obtained when self-connections are correctly retained.

This paper is organized as follows. Section 2 presents the theoretical background to NFT in matrix form, the calculation of eCMs from experimental fCMs, and the experimental dataset used in this paper to test the predictions. The results are presented in Sect. 3, where we show how the deleted self-connections in experimental fCM affect the structure of the fCM and the inferred eCMs. Methods that avoid these errors are discussed and the CMs are further analyzed using spectral methods that isolate individual eigenmode contributions to the total connectivity. The discussion and conclusions are presented in Sect. 4, where the broad applicability of the results to brain connectivity studies is emphasized.

## Methods

A key message of the present paper is that it is necessary to define all mathematical and physical quantities carefully and to respect their basic properties. Hence, in this section we briefly recapitulate the relevant aspects of linear neural field theory and how its propagators (Green functions) relate to connection matrices.

### NFT in matrix representation

Following the approach in (Robinson 2012), which pointed out that normal brain activity comprises mainly perturbations from a mean level that corresponds to a fixed point of the dynamics. Thus, we write the perturbation field of the synaptic activity of neuron population *a* at location ${\mathbf {r}}$ and time *t* as $\varPhi _{a}({\mathbf {r}}, t)$.

Since activity in a neuron population $a=1,\ldots ,P$ (where *P* is the total number of populations) is caused by neural input from populations $b=1, \ldots , P$ (including *a*) and external input $N_a$, we can write

1 $$\begin{aligned} \varPhi _{a}({\mathbf {r}}, t) =&\sum _{b} \int \int \varLambda _{ab}({\mathbf {r}}, t, {\mathbf {r}}^\prime , t^\prime )\varPhi _{b}({\mathbf {r}}^\prime , t^\prime )d {\mathbf {r}}^\prime dt^\prime \nonumber \\&+N_{a}({\mathbf {r}}, t), \end{aligned}$$

where the propagator $\varLambda _{ab}$ quantifies the activity evoked in neuron population *a* at location ${\mathbf {r}}$ and time *t* by activity afferent from neuron population *b* at ${\mathbf {r}}^\prime , t^\prime$. To preserve causality, $\varLambda _{ab}({\mathbf {r}}, t, {\mathbf {r}}^\prime , t^\prime )=0$ for $t<t^\prime$.

In CM analysis, the locations and time are usually discretized, so in Eq. (1) the integrals are replaced by sums over discrete values of ${\mathbf {r}}$ and *t*. Here, we discretize each of the *P* populations into *M* spatial regions and view synaptic activity $\varPhi _{a}({\mathbf {r}}, t)$ and external input $N_{a}({\mathbf {r}}, t)$ as *MP*-element column vectors, in which *P* groups of *M* elements each represents one population’s activity on the *M* chosen regions (Robinson 2019). Then in matrix format Eq. (1) becomes

2 $$\begin{aligned} \varPhi (t) =&\int \varLambda (t, t^\prime )\varPhi (t^\prime )dt^\prime +N(t), \end{aligned}$$

where $\varPhi$ and *N* are *MP*-element column vectors that represent activities through all spatial points for each neural population in turn, and $\varLambda$ is an $MP\times MP$ matrix. We note that each element in the matrices $\varPhi$, *N*, and $\varLambda$ includes an implicit factor that corresponds to the volume element represented by that point, and this highlights the need to use a fine discretization if the integral is to be done accurately, and also possible to determine when experimental data suffice to yield convergent results that reflect properties of the brain, rather than of discretization and thresholding (Robinson 2019). In fCM measurements, only large-scale connections of excitatory neurons via white matter are measured; hence, we only consider connectivity of the excitatory population explicitly. This means that subscripts *a* and *b* are omitted henceforth, $\varPhi$ and *N* reduce to *M*-element vectors, and $\varLambda$ is of size $M\times M$.

### Inferring eCMs from fCMs

The method of calculation of eCMs from experimental fCMs was introduced in Robinson et al. (2014). We emphasize that we only consider the symmetric case because fCMs are determined from covariances, and are symmetric by definition; they do not include information on the directionality of links.

If $\varLambda$ can be approximated as static on the timescale of cortical activity, it depends only on $t-t^\prime$. Following Eq. (2), we then have

3 $$\begin{aligned} \varPhi (t) = \int \varLambda (t-t^\prime )\varPhi (t^\prime )dt^\prime + N(t), \end{aligned}$$

where the propagator $\varLambda$ is now identified as being the spatiotemporal deCM (Robinson 2012; Robinson et al. 2014). By Fourier transforming Eq. (3) versus time, we have

4 $$\begin{aligned} \varPhi (\omega )&= [I-\varLambda (\omega )]^{-1}N(\omega ), \end{aligned}$$

5 $$\begin{aligned}&= T(\omega )N(\omega ), \end{aligned}$$

where $\omega$ is the angular frequency (this argument distinguishes Fourier transformed quantities from temporal ones, which have time as their argument), *I* is the unit matrix, and *T* is the transfer matrix that links activity $\varPhi$ to input *N*, which is approximated as white noise in the resting state, i.e., *T* is the teCM.

The teCM *T* can be expanded in a Taylor series to give

6 $$\begin{aligned} T(\omega )&= \sum ^{\infty }_{m=0} [\varLambda (\omega )]^m, \end{aligned}$$

where the powers represent successively higher-order polysynaptic paths from input locations to the cortex (Robinson 2012; Robinson et al. 2014; Mehta-Pandejee et al. 2017)

We define the fCM ${\tilde{C}}$ to be the covariance matrix of the activities (Robinson et al. 2014),

7 $$\begin{aligned} {\tilde{C}}(\tau )=\langle \varPhi (t+\tau ) \varPhi ^{T}(t) \rangle , \end{aligned}$$

where the angle brackets indicate an average over *t*. With $\tau =0$, we write Eq. (7) in terms of transfer function as (Robinson et al. 2014)

8 $$\begin{aligned} {\tilde{C}}(\omega ) = T(\omega ) T^{\dagger }(\omega ), \end{aligned}$$

in the resting state where the dagger indicates a Hermitian conjugate and is just the transpose (denoted by the superscript *T* below) at $\omega =0$ where *T* is real. For very low frequencies of fMRI, i.e., $\omega \lesssim 1~\mathrm {s^{-1}}$ (Jezzard et al. 2001; Aquino et al. 2012), we can approximate Eq. (8) as

9 $$\begin{aligned} {\tilde{C}}\approx {\tilde{C}}(\omega =0). \end{aligned}$$

We note that all matrices relevant to fMRI involve very low frequencies, so $\omega \approx 0$, and we omit this argument henceforth.

Most experimental studies use the normalized covariance matrix ${{\mathcal {C}}}$ to define the fCM; this is obtained from $\tilde{{\mathcal {C}}}$ by dividing all its elements ${\tilde{c}}_{ij}$ by the geometric mean of ${\tilde{c}}_{ii}$ and ${\tilde{c}}_{jj}$, with $i = 1,\ldots , p$ and $j = 1,\ldots , M$. If we write the elements of ${\mathcal {C}}$ as ${\tilde{c}}_{ij}$, we thus have

10 $$\begin{aligned} c_{ij} = \frac{{\tilde{c}}_{ij}}{\sqrt{{\tilde{c}}_{ii}}\sqrt{{\tilde{c}}_{jj}}}. \end{aligned}$$

If we approximate the diagonal elements by their average $\langle {\tilde{c}}_{ii} \rangle$, Eq. (10) can be simplified to

11 $$\begin{aligned} c_{ij} = \frac{{\tilde{c}}_{ij}}{\langle {\tilde{c}}_{ii} \rangle }, \end{aligned}$$

whence

12 $$\begin{aligned} C = \frac{1}{\langle {\tilde{c}}_{ii} \rangle } {\tilde{C}}. \end{aligned}$$

We emphasize that we use the normalized covariance matrix ${\mathcal {C}}$ to define the fCM. Equations (4)–(6), and (8) show that $\varLambda$, *T*, *C*, and ${\tilde{C}}$ commute. Hence using standard matrix theory for symmetric $\varLambda$, we can write

13 $$\begin{aligned} \varLambda = U L U^{\dagger }, \end{aligned}$$

where *U* is a unitary matrix whose columns are the eigenvectors of $\varLambda$, $U^{\dagger } = U^{-1}$, and *L* is a diagonal matrix of the eigenvalues $\lambda _j$ of $\varLambda$, written

14 $$\begin{aligned} L = \mathrm{diag}(\lambda _j). \end{aligned}$$

For $\omega =0$, the unitary matrices become real orthonormal matrices and the Hermitian conjugates above are equivalent to transposes.

We can also write *T* and *C* in diagonal form, analogous to Eq. (13):

15 $$\begin{aligned} T= & {} U\varTheta U^{\dagger } , \end{aligned}$$

16 $$\begin{aligned} C= & {} UK U^{\dagger } , \end{aligned}$$

where $\varTheta$ as a diagonal matrix of the eigenvalues of *T* and *K* as a diagonal matrix of the eigenvalues of *C*. Following (Robinson et al. 2014; Pinotsis et al. 2014),

17 $$\begin{aligned} \varTheta= & {} {\mathrm{diag}} (\theta _{\mathrm{j}})= {\mathrm{diag}} ([1-\lambda _{\mathrm{j}}]^{-1}), \end{aligned}$$

18 $$\begin{aligned} K= & {} {\mathrm{diag}} (\kappa _{\mathrm{j}})={\mathrm{diag}} (|1-\lambda _{\mathrm{j}}|^{-2}), \end{aligned}$$

where Eqs (17) and (18) express $\theta _j$ and $\kappa _j$ in terms of $\lambda _j$. The matrices *L*, $\varTheta$, and *K* are the deCM, teCM, and fCM expressed in terms of eigenfunctions (i.e., in the eigenfunction basis, rather than the coordinate basis defined by RoIs).

## Results

In this section, we first point out some theoretical and practical errors that result from removing self-connections in fCMs and stress that, although the mathematical cause is quite simple, the result is that serious errors have affected a whole research field, and continue to do so. We then demonstrate and illustrate the results, especially for nonmathematical audiences, and investigate how the removal of self-connections impacts the structure of fCMs and eCMs inferred from them using the publicly available NKI-Rockland experimental dataset, obtained from the USC Multimodal Connectivity Database (Nooner et al. 2012). The fCM (of size $165 \times 165$) used in this paper is based on group-average data and is a normalized covariance matrix. In each connectivity matrix, the elements are ordered so that the first 81 elements in each row and column are from the left hemisphere and the next 84 are from the right hemisphere.

A detailed description of the NKI-Rockland dataset can be found in Brown et al. (2012), so we do not reproduce this material in full here. In brief, fMRI time series were collected over several minutes per scan in voxels of $(1\text {-}2 \,\mathrm{mm})^3$. Preprocessing to remove motion artifact, reduce noise, and correct for a range of systematic effects in the experiment was then carried out. The resulting time series were clustered into 188 regions of interest (RoIs), of which 165 were cortical. The mean time series for each RoI was then calculated and correlated with the others to obtain the fCM used here, which is restricted to the cortical RoIs.

### Effect of deleting self-connections on CMs

In the NKI-Rockland fCMs, as in many others, the self-connections in the experimental fCM have been removed by setting the diagonal elements to 0. However, we know that the diagonal entries in any normalized correlation matrix are 1 by definition. In this section, we investigate how the properties of the experimental fCM are affected by deletion of the self-connections, as well as the effect of this step on the eCMs that are inferred via Eqs (13)–(18).

**Fig. 1:**
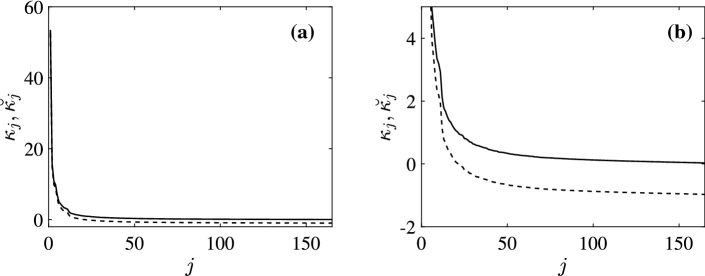
Comparison between the eigenvalues $\kappa _j$ of *C* (solid) and $\breve{\kappa }_j$ of $\breve{C}$ (dashed). **a** All eigenvalues. **b** Expanded view of eigenvalues with $\kappa _j<5$

**Fig. 2:**
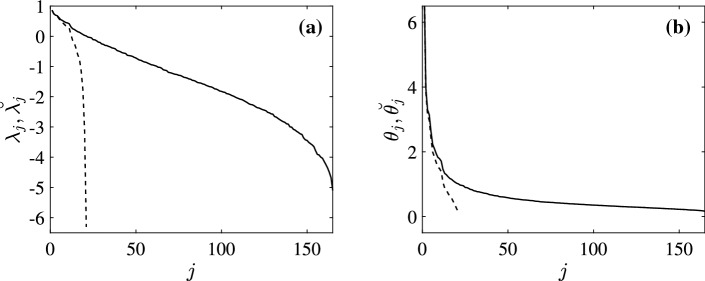
Comparison between the eigenvalues $\lambda _j$ and $\theta _j$ of $\varLambda$ and *T* calculated from *C* (solid) and $\breve{\lambda _j}$ and $\breve{\theta _j}$ of $\breve{\varLambda }$ and $\breve{T}$ of $\breve{C}$ (dashed). **a** Eigenvalues of the deCMs. **b** Eigenvalues of the teCMs

We write the normalized experimental fCMs with diagonal entries as *C*, and the fCM with diagonal entries deleted as

19 $$\begin{aligned} \breve{C} = C - I, \end{aligned}$$

where *I* is the unit matrix.

Now *C* and $\breve{C}$ commute because *I* commutes with any matrix. Hence, the three matrices share the same eigenvectors, and we can write

20 $$\begin{aligned} \breve{C} = U \breve{K} U^{\dagger }, \end{aligned}$$

where $\breve{K}$ is the diagonal matrix of the eigenvalues $\breve{\kappa }_j$ of $\breve{C}$. We know that the eigenvalues of a unit matrix are all 1, so

21 $$\begin{aligned} \breve{\kappa }_j = \kappa _j - 1. \end{aligned}$$

We use the notation $\breve{\varLambda }$, $\breve{T}$ for the deCM and teCM calculated from $\breve{C}$, respectively, and $\breve{\lambda }_j$ and $\breve{\theta }_j$ for the eigenvalues of $\breve{\varLambda }$ and $\breve{T}$, respectively.

Figure 1 compares the eigenvalues of experimental *C* and $\breve{C}$, arranged in decreasing order. The results agree with Eq. (21) to within numerical round-off errors. We notice that $\breve{\kappa }_j<0$ for $j\ge 21$, which demonstrates a fundamental error in the use of $\breve{C}$ because covariance matrices are positive definite by definition and thus must have positive eigenvalues. If we were to use $\breve{\kappa }_j$ to calculate the eigenvalues of the deCM and teCM, only 21 eigenvalues of $\breve{C}$ would potentially be available to calculate $\breve{\lambda }_j$ and $\breve{\theta }_j$ and even these are not accurate. Hence, diagonal elements of the fCM must be retained to obtain valid results.

Figures 2a, b, respectively, show the eigenvalues of the deCMs and teCMs calculated from *C* (solid) and $\breve{C}$ (dashed). We observe that use of $\breve{\kappa }_j$ leads to incorrect estimation of the eigenvalues of deCM and teCM, although the first few are approximately correct. We also note that the largest eigenvalues of the deCMs are both approximately 0.87, which is also in close agreement with prior EEG- and fMRI-based results (Robinson 2017).

**Fig. 3:**
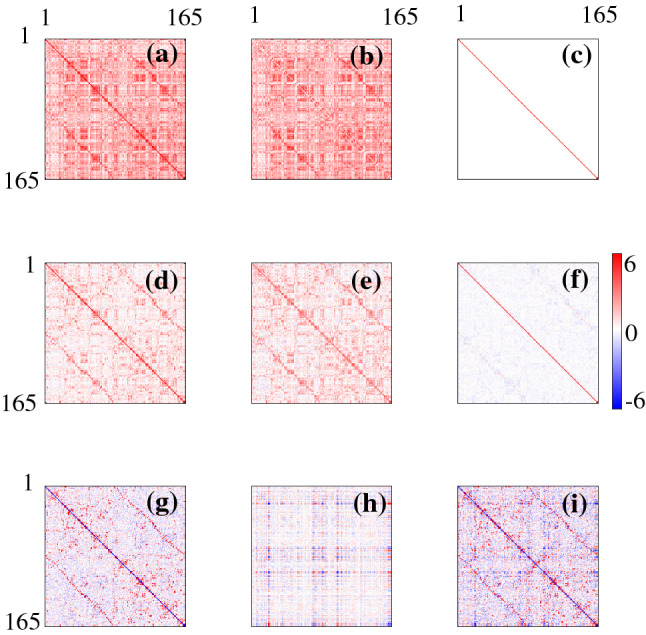
Functional CM and the corresponding inferred eCMs with the strengths of entries given by the color bar at right. **a** *C*, **b** $\breve{C}$, **c** *T*, **d** $\breve{T}$, **e** $\varLambda$, and **f** $\breve{\varLambda }$

Figure 3 compares the effect of self-connectivity on fCMs and the corresponding inferred eCMs. Comparing Figs 3a, b we notice the absence of diagonal connections in $\breve{C}$. Figure 3c shows the teCM *T* calculated from *C*, we observe structure in Fig. 3c that is similar to that in Fig. 3a, except that there is a smaller fraction of strong connections, and these are concentrated more tightly around the main diagonal and the secondary diagonals that represent interhemispheric connections between homologous regions. Some block-like structure is seen, although this is mostly an illusion that is caused by mapping the 2D cortex onto a 1D list of RoI label, it does not represent discrete modularity (Henderson and Robinson 2011)

Figure 3e shows the deCM $\varLambda$ calculated from *C*. Again, we observe that the strongest entries are on the main diagonal and secondary (interhemispheric, between homologous regions) diagonals. However, entries near the main diagonal are negative. These negative elements reflect the presence of net local inhibition that approximately balances the net excitation at longer ranges, as is required for the brain to have its observed overall marginal stability (Robinson 2017). Notably, they also demonstrate the ability of the inversion method to infer the effects of spatially unresolved structure with scales ($<1$ mm) well below those of the cortical discretization ($\sim 4$ cm). In contrast to Fig. 3a, c, many entries in $\varLambda$ are negative, this is because $\varLambda$ only quantifies the strength of direct connections between RoIs. One important point to stress here is that the net local inhibition at scales of one grid point (representing several square cm of cortex) with longer range excitation via white matter fibers must not be confused with the well-known sub-mm central excitation and surround inhibition of individual neurons. The implication is that the latter is the stronger, when integrated over distances of 1 mm or more, and that it must then be balanced by even longer-range excitation to achieve the observed near-criticality of the cortex.

We next examine the CMs calculated from $\breve{C}$ in Fig. 3b. We see that the incorrect estimation of the eigenvalues $\breve{\theta }_j$ leads to incorrect estimation of $\breve{T}$. Although the diagonals and block-like structures appear in $\breve{T}$, the connectivity strength, especially the entries in the main diagonal are significantly reduced in $\breve{T}$. Figure 3f shows $\breve{\varLambda }$, which shows almost no sign of the main structures seen in $\varLambda$. This is because most of the negative eigenvalues in $\varLambda$ are not captured in $\breve{\varLambda }$. Thus, our results show that the missing self-connections in the fCM greatly affect the estimated connections within and between hemispheres in the inferred eCMs and thus must be retained to obtain the accurate results. At one level this is to be expected, but the extent of the changes is surprising, given how small a proportion of the connections are deleted in the fCM—only 0.6% in the present case.

**Fig. 4:**
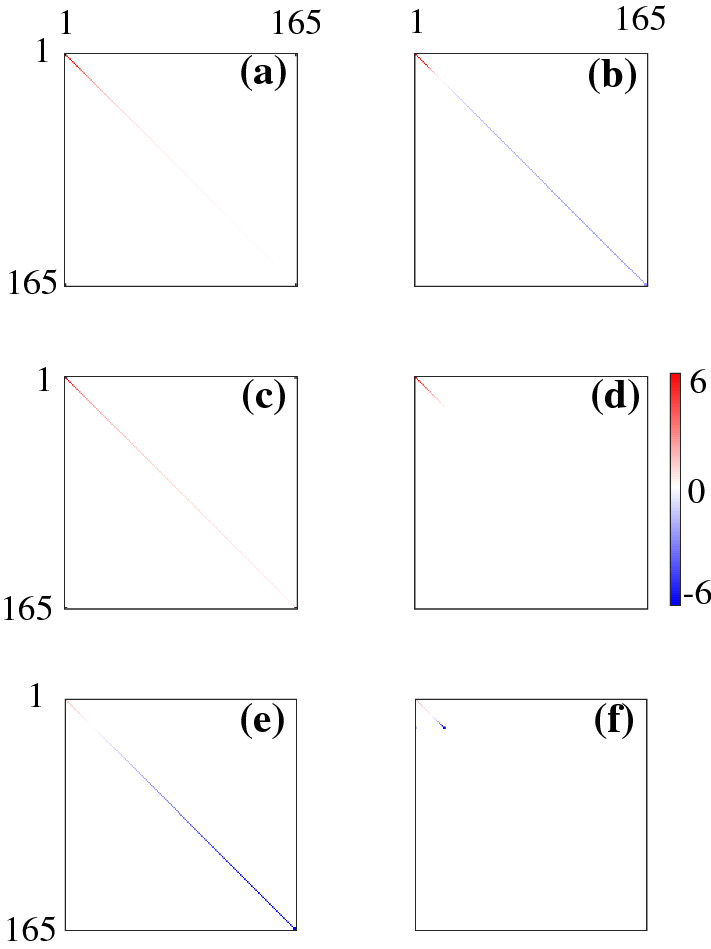
Diagonalized fCMs and the corresponding inferred eCMs with the strengths of entries given by the color bar at right. **a** *C*, **b** $\breve{C}$, **c** *T*, **d** $\breve{T}$, **e** $\varLambda$, and **f** $\breve{\varLambda }$

From the theoretical analysis, we know that the eigenmodes of the fCM and the inferred eCMs are the same. By using eigenmode analysis and spectral method we can write the connectivity matrix in diagonal form. Figure 4a, b highlights the dramatic simplification relative to Fig. 3a, b by showing the diagonalized fCMs *K* and $\breve{K}$, respectively. The entries in Fig. 4a are all positive because *C* is a covariance matrix and thus can have only positive eigenvalues. However, in Fig. 4b, only 21 eigenvalues are positive, as was discussed in Sect. 3.1. Figure 4c shows the diagonalized *T* (i.e., $\varTheta$) calculated via Eq. (17), again showing an enormous simplification with all eigenvalues positive. Figure 4e shows the diagonalized $\varLambda$ (i.e., *L*) calculated using Eq. (13). In this case, we observe negative $\lambda _j$ at large *j* in Fig. 4e. Figure 4d, f shows the diagonal matrices $\breve{\varTheta }$ and $\breve{L}$, respectively. Since only 21 eigenvalues in $\breve{C}$ can be used in calculating $\breve{T}$ and $\breve{\varLambda }$, we only observe limited eigenvalues in both figures, which leads to the incorrect estimation of eCMs shown in Fig. 3. We note that NFT in a spherical geometry (Robinson et al. 2016; Robinson 2019) implies

22 $$\begin{aligned}&\lambda _j \sim -j, \end{aligned}$$

23 $$\begin{aligned}&\theta _j \sim j^{-1}, \end{aligned}$$

and

24 $$\begin{aligned} \kappa _j \sim j^{-2}, \end{aligned}$$

at large *j*, shown in Figs. 1 and 2, all of which are consistent with the results shown in Fig. 4. Therefore, $\theta _j$ and $\kappa _j$ converge to 0 as *j* increases, whereas $\lambda _j$ grows. Thus, only *C* and *T* are expected to give diagonal forms that are dominated by just a few modes, in accord with the results in Fig. 4. The teCM *T* is far more important dynamically than $\varLambda$ because it includes all connections, not just direct ones (Robinson 2012; Robinson et al. 2014, 2016).

### Modal analysis of CMs

Using the above data, we now investigate the contributions of individual modes to the CMs *C* and *T*, which have the most compact forms. Previous work has shown the lowest terms to be dominant (Robinson et al. 2016), but the form of the individual contributions to the CMs has not been previously studied. The contributions of the *j*th eigenmode to *C* and *T* are

25 $$\begin{aligned} C_j = U K_j U^{\dagger }, \end{aligned}$$

and

26 $$\begin{aligned} T_j = U \varTheta _j U^{\dagger }, \end{aligned}$$

respectively, where

27 $$\begin{aligned} K_j = \mathrm{diag}(0,\ldots ,0,\kappa _j,0\ldots ,0), \end{aligned}$$

and

28 $$\begin{aligned} \varTheta _j = \mathrm{diag}(0,\ldots ,0,\theta _j,0\ldots ,0). \end{aligned}$$

We also define the partial sums of the first *m* eigenmode contributions to *C* and *T* to be

29 $$\begin{aligned} S_m = \sum _{j=1}^m C_j, \end{aligned}$$

and

30 $$\begin{aligned} V_m = \sum _{j=1}^m T_j, \end{aligned}$$

respectively.

**Fig. 5:**
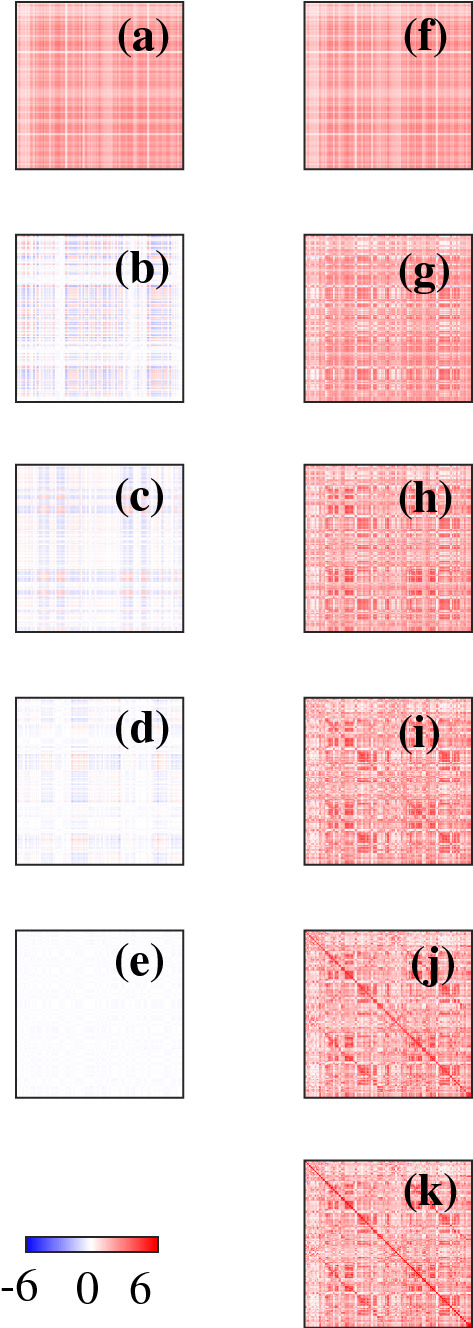
Functional connectivity matrix in modal analysis with the strengths of entries given by the color bar at bottom left. **a**–**e** show modal contributions $C_1$, $C_2$, $C_3$, $C_5$ and $C_{20}$, respectively; **f**–**j** show partial sums $S_1$, $S_2$, $S_3$, $S_5$ and $S_{20}$, respectively; and **k** shows *C*

Figure 5 shows modal contributions to *C* and their partial sums. Figure 5a–e shows the contributions $C_1$, $C_2$, $C_3$, $C_5$ and $C_{20}$, respectively, while Fig. 5f–j shows the corresponding partial sums $S_1$, $S_2$, $S_3$, $S_5$, and $S_{20}$, and Fig. 5(k) shows *C* for comparison. The first mode, shown in Fig. 5a, is approximately spatially uniform and has no negative entries, which is consistent with the previous results that this corresponds to a uniform mode (Robinson et al. 2016; Gabay and Robinson 2017). The next modes have approximately equal numbers of positive and negative entries, consistent with their mean values being zero because of orthogonality to the lowest, uniform mode. These contributions are spatially nonuniform because their spatial eigenmodes have nodal lines that divide positive from negative regions (Robinson et al. 2016; Gabay and Robinson 2017). The size of these contributions drops rapidly from $C_1$ to $C_{20}$ as the eigenvalues decrease. As shown in Fig. 5g–i, the block-like structures start to appear in $S_2$, the diagonals start to appear in $S_3$ and most of the strongest connectivity is already present in $S_5$, in accord with just the first few eigenmodes being dominant (Robinson et al. 2016). Comparing Fig. 5j, k, we observe that the connectivity strength is almost the same for $S_{20}$ and *C*. This shows that to obtain the full sharpness and strength of the diagonals, requires superposition of around 20 or more modes to obtain sufficient spatial localization.

**Fig. 6:**
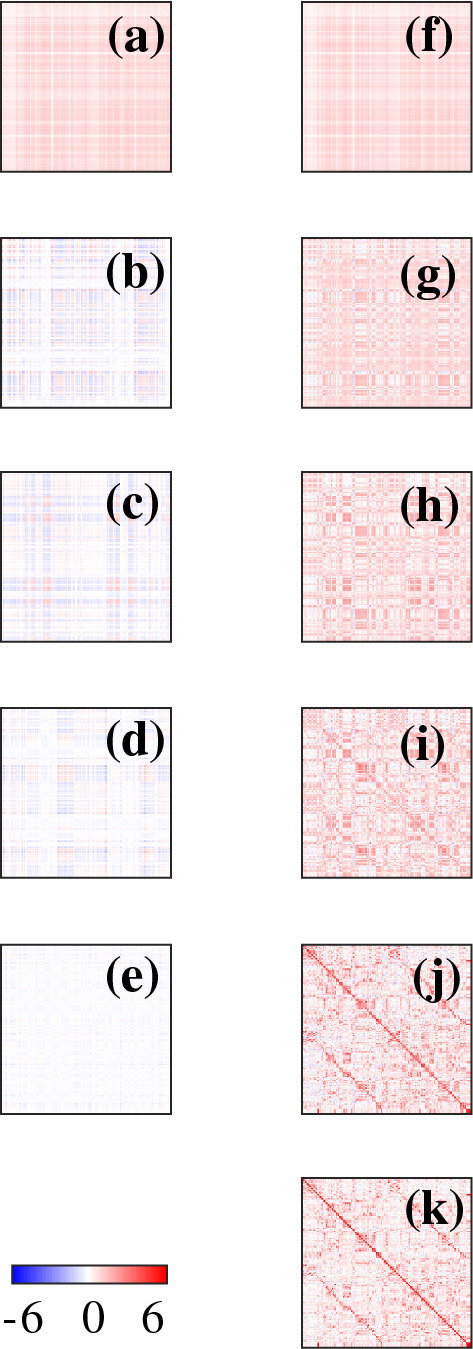
Total effective connectivity matrix in modal analysis with the strengths of entries given by the color bar at bottom left. **a**–**e** show $T_1$, $T_2$, $T_3$, $T_5$ and $T_{20}$, respectively; **f**–**j** show $V_1$, $V_2$, $V_3$, $V_5$ and $V_{20}$, respectively; and **k** shows *T*

Figure 6 shows modal contributions to *T* and their partial sums. Figure 6a–e shows modal contributions $T_1$, $T_2$, $T_3$, $T_5$ and $T_{20}$, respectively, Fig. 6f–j shows partial sums $V_1$, $V_2$, $V_3$, $V_5$ and $V_{20}$, respectively, and Fig. 6k shows *T*. As for $C_1$, we find that $T_1$ in Fig. 6a is approximately uniform. Indeed, the structure of each eigenmode contribution is the same as for *C* because *C* and *T* have the same eigenvectors, but the weights decrease more slowly in *T* because of the slower decrease of its eigenvalues. Comparing Fig. 6j–k, we again observe that accurate representation of the main diagonal requires retention of more modes than other parts of the matrix.

## Conclusion

The importance of retaining self-connections in experimental fCMs and the eCMs inferred from them has been investigated using methods from spectral analysis and neural field theory. We have introduced new methods to explore the connectivities and their effects on the relationships between functional and effective CMs using eigenmode analysis and spectral methods through which we also represent the brain connectivity in a compact diagonal matrix form. The results have been illustrated and verified using NKI-Rockland data, which underline the need to adopt these new methods to ensure the accurate results. The main findings are:

(i) The near-universal step of in published studies, deleting diagonal entries from functional CMs defined by activity covariances, is invalid. It violates fundamental mathematical requirements on covariance matrices and invalidates the physical relationships between functional and effective CMs, as detailed in the following points. Again, we stress that the fact that there is a change of some kind is mathematically obvious and the central purpose of the paper is thus not merely to establish its existence, but to explore its nature and the resulting effects on both the fCM and, more importantly, on the effective CMs that are inferred from it via NFT.
(ii) The analytical and numerical results show that all eigenvalues of *C* decrease by 1 after deleting the self-connections. Therefore, most of the eigenvalues of the fCM without self-connections become negative, which is impossible for a covariance matrix, and indicates a fundamental error. These negative values cannot be used to validly calculate eCMs (Robinson et al. 2014). Thus, it is mathematically essential for self-connections to be retained.
(iii) Because the fCM, deCM, and teCM are symmetric and commute, they share the same eigenvectors and can be represented in closely related diagonalized forms. However, unless self-connections are retained, the inferred eCM structures are severely affected, especially for the deCM, whose diagonals and block-like structures are not captured. These changes are fundamental and disproportionate to the small number of connections deleted. Deletion of fCM self-connections also implies widespread long-range differences in the corresponding effective connectivities and removes the ability to infer short-range net inhibitory connections at subgrid scales. (Note that these include the sub-mm Mexican-hat structure of very short range excitatory connections with inhibitory surround, plus longer excitatory connections out to $\sim 2$ cm but within the same region of interest.)
(iv) We have decomposed the fCM and teCM via eigenmode analysis, retaining self-connections and confirming that the first few eigenmodes suffice to reproduce the main features of *C* (Robinson et al. 2014, 2016) and *T*. We also showed that to accurately represent diagonal entries of the fCM, only around 20 eigenmodes are needed for NKI-Rockland data with $N=165$ regions of interest. This contrasts with the $N(N+1)/2=13 695$ entries that are required in a conventional representation.
(v) The deCM $\varLambda$ has more large entries in its diagonal representation than do *C* and *T*, because $|\lambda _j|$ increases at large *j*, but $\varLambda$ only summarizes direct connections between RoIs, whereas *T* is the quantity of direct dynamical relevance.

In summary, we have investigated the importance of retaining self-connections when analyzing the experimental fCM and the corresponding inferred eCMs. We have shown that the self-connections in the fCM play essential mathematical and physical roles in the correspondence between fCMs and eCMs and thus cannot be deleted from experimental data if one wishes to have mutual consistency between these quantities; this is contrary to their usual removal. We note that this does not make a difference in case-control comparisons, where the self-connection terms cancel, but inferred relationships between fCMs and eCMs in existing studies in which diagonal fCM elements have been deleted should be reviewed. This underlines the need to respect and preserve the formal properties of quantities being measured (in this case, the covariance matrix) to avoid the potential for mathematically invalid analysis steps. We have also shown that the methods discussed here deliver compact spectral representations of CMs and verify that these can greatly simplify treatment of brain connectivity, thus promising new and more tractable analyses and insights. These latter results have been illustrated in familiar CM form to emphasize their utility.
